# Evaluating statistical approaches to define clonal origin of tumours using bulk DNA sequencing: context is everything

**DOI:** 10.1186/s13059-022-02600-6

**Published:** 2022-02-02

**Authors:** Tanjina Kader, Magnus Zethoven, Kylie L. Gorringe

**Affiliations:** 1 Peter MacCallum Cancer Centre, 305 Grattan St, Melbourne, 3000 Australia; 2 The Sir Peter MacCallum Department of Oncology, The University of Melbourne, Parkville, 3010 Australia

## Abstract

Clonal analysis of tumour sequencing data enables the evaluation of the relationship of histologically distinct synchronous lesions, such as co-existing benign areas, and temporally distinct tumours, such as primary-recurrence comparisons. In this review, we summarise statistical approaches that are commonly employed to define tumour clonal relatedness using data from bulk DNA technologies. We discuss approaches using total copy number, allele-specific copy number and mutation data, and the relative genomic resolution required for analysis and summarise some of the current tools for inferring clonal relationships. We argue that the impact of the biological context is critical in selecting any particular approach, such as the relative genomic complexity of the lesions being compared, and we recommend considering this context before employing any method to a new dataset.

**Supplementary Information:**

The online version contains supplementary material available at 10.1186/s13059-022-02600-6.

## Background

A central dogma of oncology is that clonal expansion, in which tumours arise from a genetically altered cell and develop into an invasive cancer, occurs in a stepwise manner with sequential somatic cell mutations along with subclonal selection. This revolutionary perspective described by Peter Nowell in 1976 meant that cancer is indeed an evolutionary process [1]. Early molecular studies attempted to elucidate tumour development from premalignant lesions to carcinoma by their genetic similarities [2, 3] using various technologies including cytogenetic analysis, Loss of Heterozygosity (LOH) studies, and mutations in known oncogenes/tumour suppressor genes (*KRAS* or *TP53*).

In order to understand cancer progression from pre-malignant lesions or the relationship of clinically identified “paired” tumours, the ability to accurately estimate clonal relatedness (i.e. whether paired tumours share a common ancestor) is crucial. Different statistical and analytical approaches have been used to investigate clonal relationships from data generated by multiple techniques using bulk DNA [4–8]. In addition, deep sequencing has revealed the co-existence of major and minor clones within the same tumour; we also explore this intra-tumour heterogeneity (ITH) as it is a very important phenomenon in the clinical context.

This Review summarises the evolution of these statistical approaches and discusses the strengths and limitations of each approach. We focus on bulk technologies (not single cell) for DNA analyses, firstly considering the total and allele-specific copy number data that can be obtained from both pre-next generation DNA sequencing (NGS) employing microarrays (CGH arrays, SNP arrays) and current low-resolution NGS technologies (lower depth sequencing, targeted sequencing panels). Given the limitations of using copy number data alone, we will also consider the impact of including somatic point mutation data from NGS technologies when determining clonal relatedness. We consider approaches that are suitable using low depth NGS data as well as address the importance of high depth NGS technologies and how subclonal reconstruction and phylogenetic analyses have changed our understanding of cancer evolutionary models.

Finally, given the limitations of existing approaches, especially for data derived from lower resolution technologies, we consider the appropriateness of statistical methods based on the biological context. We will discuss premalignant lesions and metastasis settings and how different contexts might influence the selection of statistical methods.

## Estimating similarities from somatic copy number alteration data

**Fig. 1:**
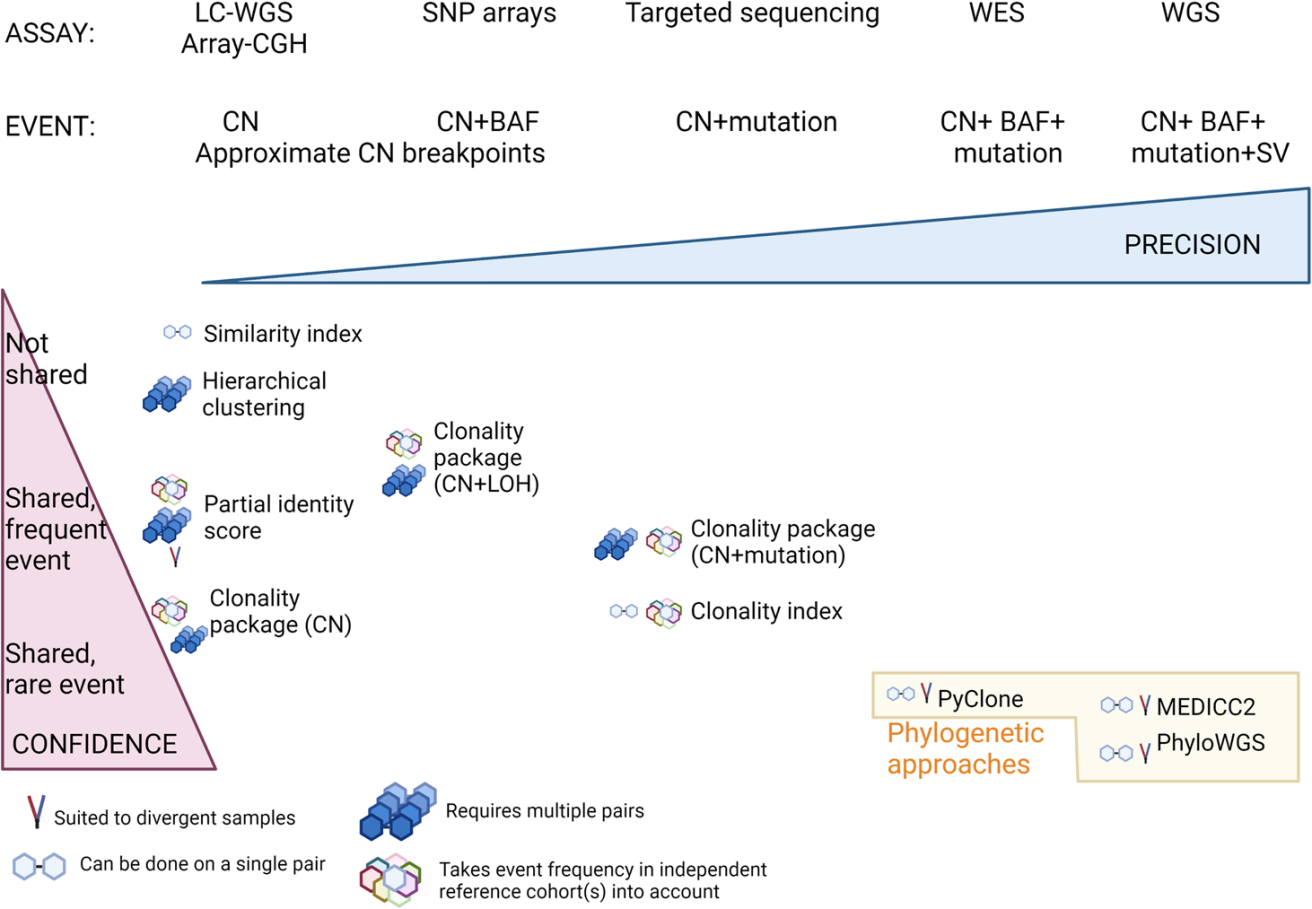
Summary of clonality approaches illustrating their features in relation to the type of genetic event. Clonality methods are placed horizontally relative to the minimal assay type that can be used and vertically by the relative confidence each method provides in the clonal relationship. CN, copy number; SV, structural variation; BAF, B-allele frequency and the assay method; LC-WGS, low coverage whole genome sequencing; CGH, comparative genomic hybridisation; SNP, single nucleotide polymorphism; WES, whole exome sequencing; WGS, whole genome sequencing

Since aneuploidy is a hallmark of many cancers, somatic copy number alterations (SCNA) are a valuable genetic event for assessing clonal relationships between samples (Fig. 1). SCNA are generally assessed using two broad approaches: total SCNA, or assessed from allele- and haplotype-specific SCNA. Recent studies also show that with high depth sequencing data, evolutionary methods can be utilised to reconstruct tumour phylogenies to understand the complex ITH of a single tumour. Here in the first part of the review, we will discuss the statistical approaches applicable to low resolution technology as well as the evolutionary methods applicable to high depth sequencing methods (Fig. 1, Table 1).

**Table 1:** Summary of the types of technologies used in clonality analysis

Methods	Depth/resolution	CNA	BAF	Variant detection	Subclonal reconstruction	Comment
CGH arrays	Low: 5–10 Mb	✓	**x**	**x**	**x**	Suitable for clonality analysis in tumours with high SCNA
Low coverage WGS	Low: <4×	✓	**x**	**x**	**x**	
SNP arrays	300 K–1 M SNPs	✓	✓	**x**	**x**	Suitable for clonality analysis in tumours with high and low SCNA
Targeted sequencing panel	High depth (>200×) but only on target regions	✓	**x**	✓	✓	SCNA called from off-target sequence suited to clonality analysis but BAF may be unreliable with so few genes; phylogenetic analysis is possible with ultra-deep sequencing (>500×) using TRONCO but limited power due to selective genomic markers [9]
Low coverage WES	30–60×	✓	✓	✓	**x**	Read depth might be too low for subclonal analyses but suited to clonality analyses of paired/multi-region samples. Needs matching normal DNA for maximum power.
High coverage WES	High: >60×	✓	✓	✓	✓	Powerful for clonality analyses but needs matching normal DNA. Low purity might interfere with the estimation of subclonal SCNA especially at lower depth.
WGS	High breakpoint resolution but depth can be low >30×	✓	✓	✓	✓	
Single cell sequencing	Low individual cell resolution	✓	✓	**x**	✓	Individual cell allele-specific CN for deep subclonal reconstruction [10, 11]; mutation calling still challenging due to allelic dropout at the individual cell level.

*CGH* comparative genomic hybridisation, *SNP* single nucleotide polymorphism, *WES* whole exome sequencing, *WGS* whole genome sequencing, *SCNA* somatic copy number alterations, *BAF* B-allele frequency

### Use of only total SCNA concordance to derive the similarity profile of a tumour pair

An early approach to assessing clonality using CGH arrays was performed by Waldman et al. [4] based on the overall SCNA concordance of primary and recurrent ductal carcinoma in situ (DCIS) breast lesions (*n*=18). This approach was followed by Biermann et al. [12] later on, who assessed the similarity index (SI):

$$ \mathrm{SI}=\mathrm{Ns}/\left(\mathrm{Ns}+\mathrm{Nu}+\mathrm{No}\right). $$

Here SI = Similarity Index, Ns=shared events, Nu=unique events and No=opposite events between pairs. Notably, for this index, only one chromosomal change per chromosome arm is considered as a genetic event (gain/loss).

Hierarchical clustering, for example using the Pearson coefficient, is another commonly employed method to investigate the genetic similarities based on overall SCNA [13, 14]. An unsupervised analysis is employed whereby complete linkage (cluster method) and Pearson correlation (distance metric) are used to relate the similarity of samples and a dendrogram generated to depict similarity (shorter branched cluster) or disparity (longer branched) of genomic changes among tumours. Thus, clustering together of two tumours from the same patient indicates the relative similarity of the pairs, but cannot determine the statistical confidence of these relationships and cannot be applied to a single pair alone.

These fairly low resolution approaches may not identify the true extent of clonal relationships or may give an equivocal result, especially when there are more private rather than shared events, as can occur in distantly related lesions. In addition, they might falsely call clonality if specific events are shared by chance, such as gains or losses that occur at commonly involved loci.

The analysis by Waldman et al. raises the question of whether total SCNA alone is sufficient to estimate clonality. Importantly, this study investigated recurrent breast tumours after ductal carcinoma in situ (DCIS), genetically advanced lesions that had a very similar frequency of SCNA as IBC [15, 16]. Thus, it is possible that some pairs would appear to show clonal relatedness by chance. For rare tumours this may not be an issue; unless the individual has a genetic predisposition to a particular rare tumour type, the weight of clinical evidence would always strongly suggest a recurrence. However, for tumours such as breast or colorectal cancer, there is a significant probability that independent tumours share SCNA at commonly affected loci by chance.

### Total SCNA approaches incorporating breakpoint locations

The lack of precision offered by considering only broad SCNA can be partially compensated for by including information on copy number breakpoints. A comparison between the two approaches (overall SCNAs *vs* shared breakpoints) was carried out by Bollet et al. [6] using fresh frozen invasive ipsilateral breast cancer pairs (recurrence/new primary) (*n*=22) using SNP arrays. Samples had to have ≥50% cancer cell cellularity for maximal sensitivity and the location of breakpoint had to be precise down to the SNP probe for a pair to be called genetically related. For example, the breakpoints of *PTEN* losses differed among patients, demonstrating the power of using of precise breakpoint(s) over SCNA alone.

A “partial identity score” (PS) was developed by taking into account the frequency of each shared breakpoint within a set of control samples. Therefore, the final PS was:

$$ {\mathrm{PS}}_{\mathrm{E}1,\mathrm{E}2}=\sum {\left(1\hbox{-} {\mathrm{F}}_{\mathrm{k}}\right)}^2/\frac{1}{2}\times \left[\sum {\left(1\hbox{-} {\mathrm{F}}_{\mathrm{k}}\right)}_{\mathrm{E}1}+\sum {\left(1\hbox{-} {\mathrm{F}}_{\mathrm{k}}\right)}_{\mathrm{E}2}\right] $$

Here, F_k_ is the frequency and E1 and E2 are the tumour pairs.

The strength of this score is the weighting applied based on the frequency of the shared breakpoint: less frequent shared breakpoints are less likely to happen by chance in the pairs. Another strength of this score is the specificity of the cut-off value. The threshold was calculated based on calculating the 462 possible “artificial pairs” from the 22 sample pairs (i.e. 22 patients were artificially paired with each of the 21 other patients). If the PS is only higher than the upper 5th percentile in the distribution of the artificial pairs, then the null hypothesis (there is no partial identity between the tumour pairs) was rejected (i.e. the pairs are genetically related to each other). The higher the score is, the more likely the IBC is a true recurrence. To make this score statistically sound, 1000 random extractions were performed in these 22 pairs to confirm the status of all pairs using the threshold. This approach of using the frequency of shared genetic events in a “control*”* group as well as calculating “artificial pairs” was later utilised by Ostrovnaya et al. while developing the Clonality R Package. The disadvantage of this approach is that the threshold has to be recalculated with the addition of samples and that the threshold cannot be compared across different studies. In this study, the derived PS both outperformed overall SCNA analysis by hierarchical clustering, based on the higher concordance with clinical recurrence definition. However, one case was re-reviewed by a pathologist after getting a discordant result between using PS (true recurrent) and clinical definition (new primary based on histopathological differences), which resulted in reclassifying the primary with the presence of a minor component of micropapillary carcinoma that was initially overlooked. This case shows us a great example why defining “true recurrence” based on only subjective histopathological review instead of shared genetic events could lead to different information relevant to the patient.

In summary, Bollet et al. concluded that using precise breakpoints was more accurate and meaningful than the clinical definition. One thing to consider though was that their samples were from fresh frozen tissues with high-quality CN data enabling precise breakpoints to be determined. It is more technically challenging to get the precise breakpoint(s) on DNA from FFPE specimens [17, 18]. The resolution and inherent noise in each assay, as well as the quality of individual sample data must be taken into account when applying a shared breakpoint approach. Noisy data can lead to inexact or over segmentation, thus comparing breakpoints without taking this inaccuracy into account could be misleading, i.e. the breakpoints may in fact be the same, but miscalled in one or both samples. However, if some leeway is provided in calling breakpoint similarity, false positive clonality calling based on breakpoints is only likely to be an issue in regions with frequent breakpoints, such as centromeres, which have additional challenges in terms of accuracy due to the presence of repetitive sequences [19].

### Estimating similarity from allele-and haplotype-specific SCNA

**Fig. 2:**
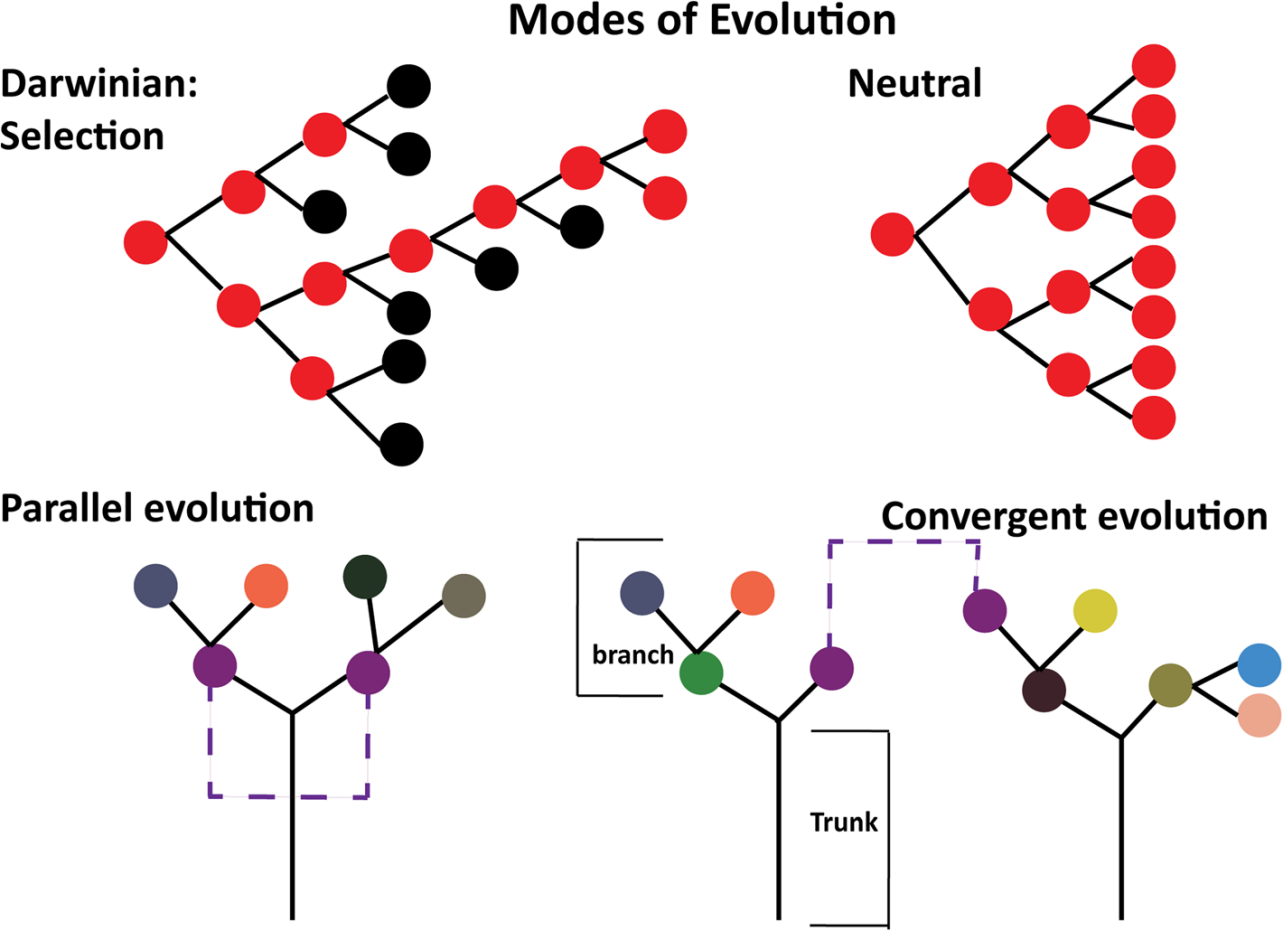
Summary of evolutionary models. This figure depicts different aspects of cancer evolution and is adapted from [20, 21]. In parallel evolution, the tumour arises from a single cancer-initiating cell (at the base of the trunk) in the same patient, whereas in convergent evolution, phenotypically similar tumours arise from different cancer-initiating cells in the same or different patients. Purple circles depict tumour subclones with a similar phenotype

In many cancer types, both parallel and convergent evolution have been detected (Fig. 2). In the parallel model, a patient tumour from one initiating cell and sharing early truncal events gives rise to subclones that then progress independently, but have a similar phenotype due to genetic events affecting similar pathways. Breast cancer has been described with a parallel evolutionary model by multiple studies. A convergent model describes different initiating cancer cells that acquire the same or highly similar genetic alterations and phenotype [20, 22]. Germline driven *VHL*-associated renal cell carcinoma [23] and squamous cell carcinoma [24] were both shown to be convergent, whereby even independent tumours carry similar genetic alterations overall. This convergence can include SCNA since both tumours are evolving in the same host with the same microenvironment and similar selection pressures. Thus, when only lower resolution data is available, any approaches taking into account only total SCNA might give a strong clonality signal regardless of the true relationship. This issue is particularly problematic for cancers with low chromosomal instability such as reported for ~ 30% of IBC (METABRIC study IntClusters 3 and 4 - the “SCNA devoid” subgroups) [25]. However, including allele-specific copy number (ASCN) can improve the detection of clonal relatedness versus independent convergent evolution.

**Fig. 3:**
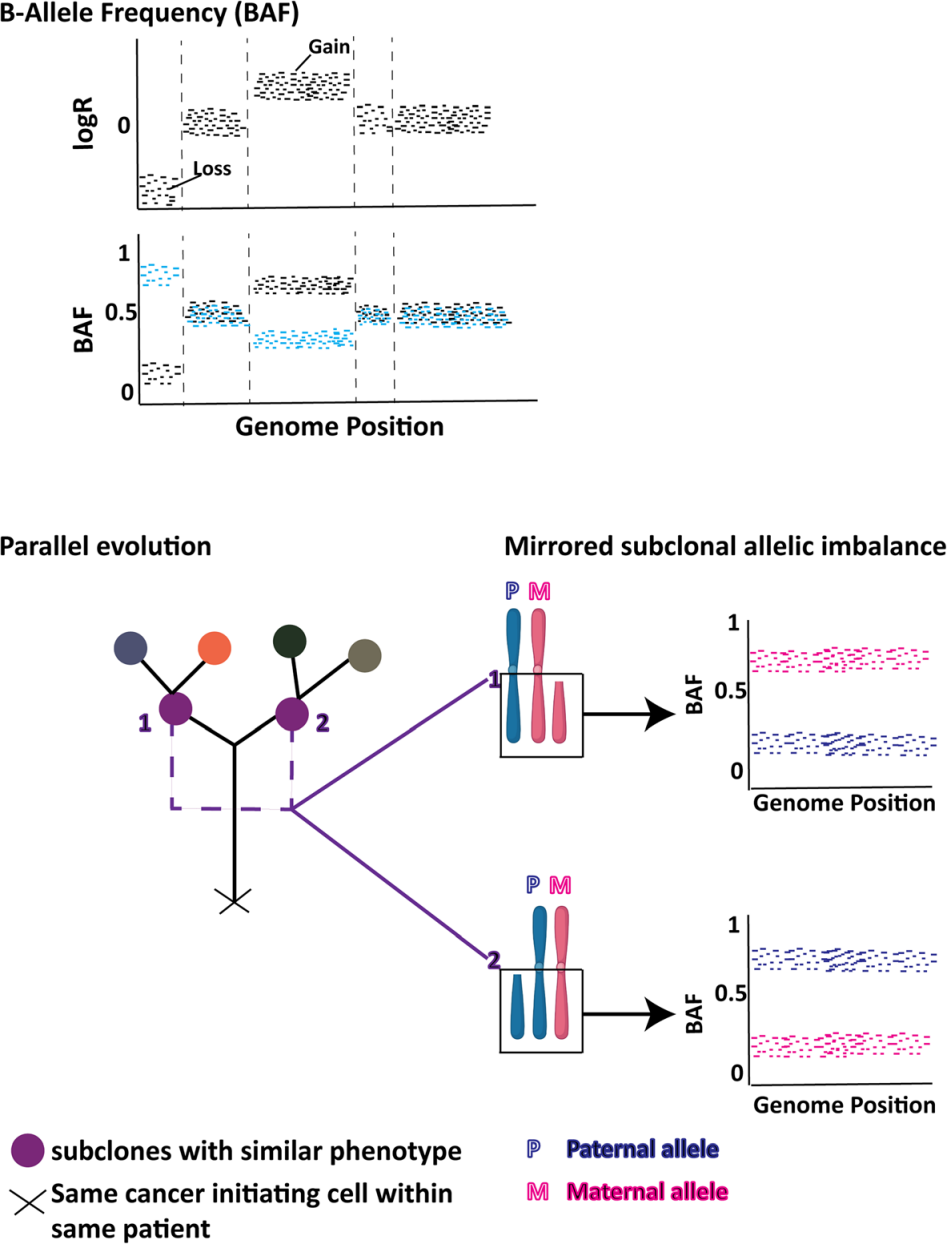
Example of allele-specific copy number changes. The top panel shows an example of copy number changes with B-Allele Frequency. The bottom panel shows an example of mirrored subclonal allelic imbalance. This term is used when within the same tumour, one subclone has gained or lost the maternal allele but the other subclone has gained or lost the paternal allele of the same chromosome, indicative of a parallel evolutionary events. Only high depth sequencing data can estimate with this level of resolution

The approach of Bollet et al. [6] was developed into a statistics-based clonality analysis (Clonality: an R package) incorporating SCNA and B-allele frequency (BAF) [26, 27]. BAF (i.e. the allelic ratio of heterozygous SNPs in a tumour) (Fig. 3) is more powerful than the copy number alone when estimating clonality. The BAF can also identify copy neutral loss of heterozygosity (LOH) events. This package aimed to test whether *relatively close* SCNA can be statistically representative of the *exact* changes shown by Bollet et al.*,* in situations where precision matches within a chromosomal arm are not feasible due to the low resolution and technical noise in the assay, for example CGH arrays, targeted sequencing or low-coverage WGS on FFPE samples. In fact, only one prominent SCNA per chromosome arm was used for this analysis, as described earlier for SI [5]. Segmentation of the CGH data allows selection of only one prominent SCNA/chromosome. No separate reference group of the same disease was used, instead, they created a reference distribution of genetic changes using the available data set being tested [27]. This reference distribution was used to create *P* values for the tumour pairs. This feature is an advantage for tumours where no independent data sets are available, but is problematic if only a small group of samples is being tested. Since this package was designed to increase the influence of shared *infrequent* CN changes, it is important to make sure that germline variants are removed prior to analysis. Otherwise, those small changes will lead to overcalling of clonality.

For copy number analysis, the final statistic for this package is LR2 = LR+LR1, whereby LR calculates frequencies of gains/losses for each chromosome arm based on the dataset being tested and LR1 is calculated based on the concordance of the gain/loss/normal profiles within each arm between pairs, taking discordant events into account. While LR takes into account any chromosomal changes including whole arm changes, LR1 is exclusive of whole arm gain/losses due to the ineffective measurement of the *exact* end point of frequent whole arm changes. Combining these two are important since common concordant events could be occurring in paired tumours by chance. For BAF, the LR test output provides the *P* value based on the concordant losses (i.e. LOH of the same allele) in tumour pairs, assuming that allele 1 and 2 have an equal chance of loss in any given pair. This test also uses the frequency of LOH events from the reference distribution. Thus, concordance of infrequent LOH between a pair will give stronger evidence towards clonality than a frequent event.

All of the statistical analyses of this package were tested on paired invasive tumours to identify whether the recurrence/metastasis is a new tumour or related to the primary tumour. Therefore, using the same testing dataset as the reference distribution is statistically justifiable because invasive tumours are genetically very advanced lesions with a high number of SCNA. However, this feature may not be suitable in some biological contexts, which will be discussed in more detail later in this review.

With higher depth multi-region sequencing, allelic imbalance from the BAF is a very powerful measure for subclonal reconstruction for both paired tumours and within a single tumour. For example, in a cohort of early-stage non-small cell lung cancer (NSCLC) different subclones from different branches of the same phylogenetic tree (i.e. from the same tumour) had gain or loss of different alleles of the same chromosome, termed “mirrored subclonal allelic imbalance” (Fig. 3) [22]. In addition, using multi-sample phasing, haplotype-specific CN was achieved with even higher resolution [28]. With low resolution data it might be impossible to get the subclonal structures of a given tumour. ITH will be underestimated and deriving an evolutionary model difficult; nonetheless, any BAF data will increase the ability to determine clonal relationships between distinct lesions.

### Evolutionary methods for SCNA

If high depth sequencing data is available, then using evolutionary methods is more powerful than concordance approaches to understand ITH within a single tumour or when estimating clonal relatedness between tumour pairs. Cancer phylogenetic trees constructed by such methods contain a trunk (i.e. all clonal descendants of the initiating tumour cell harbour these genetic alterations) and branches (i.e. descendant subclonal populations carrying additional genetic alterations) (Fig. 2).

Campbell et al. showed the presence of subclonal genetic alterations in a proportion of chronic lymphocytic leukaemia, indicating that cancer had undergone branched evolution [29], and phylogenetic reconstruction of other tumour types has confirmed this finding. Branched evolution was further characterised as either parallel or convergent evolution (Fig. 2) [20]. The composition of clonal and subclonal populations are clinically very relevant. The presence of complex subclonal populations indicates an aggressive tumour with increased capacity to metastasise, evade the immune system and develop drug resistance.

Several phylogenetic analyses for subclonal reconstruction have been developed and modified over time and are detailed in reviews by Beerenwinkel et al. [30] and Schwartz & Schaffer [31]. Large cohort studies such as TRACERx have confirmed genome doubling as one of the most common clonal events linked to worse prognoses in 87% of the tumours across 22 different cancer types [22, 28]. This Whole Genome Doubling (WGD) event leads to ongoing chromosomal instability reflected by diverse subclonal structures and extensive ITH compared to non-WGD tumours [22, 28, 32]. Recent studies using pan-cancer multi-region sequencing and WGS showed that these late subclonal events can influence the progression of invasive cancer to metastasis [28, 33]. Tetraploid cells generated by WGD have even been observed as an early event when progressing from premalignant to malignant [34–36]. Therefore, a tool that can derive a phylogenetic tree based on both SCNA and WGD can determine early clonal events while estimating diversity within a single tumour. Such tools can also estimate shared clonal and subclonal SCNA between a tumour pair to determine clonal relatedness.

A phylogenetic tree based on SCNA changes can be derived utilising MEDICC (Minimum Event Distance for Intra-tumour Copy-number Comparisons) [37]. This tool has been used on WGS data to explore spatial and temporal clonal heterogeneity and its influence on overall survival of high-grade serous ovarian cancer patients [38] as well as deriving a *big bang model* of evolution in colorectal tumours [39]. MEDICC involves three steps: (i) allele-specific assignment of major (i.e. the larger) and minor copy numbers, (ii) estimating evolutionary distance between samples followed by tree interference (the tree topology) and (iii) reconstructing the ancestral genome based on the number of events between genomes to determine the final branch lengths [37]. Recent studies suggest that detecting subclonal allelic imbalance using multi-sample phasing will improve the reconstruction of the phylogenetic tree [22, 28], particularly if mirrored allelic imbalance is present (loss of maternal allele in one subclone and paternal allele in another). MEDICC2 incorporates WGD, while also taking parallel evolution into account through the use of the Minimum-Event Distance [11]. This measure determines the shortest genetic event pathway (differences in gains, losses, WGD and allele-specific events) between two genomes. The allele-specific SCNA profile is needed to be pre-phased using multi-sample reference phasing [28] or has undergone evolutionary phasing to determine the final haplotype prior to computing the Minimum-Event Distance by MEDICC2. MEDICC2 accurately identified WGD events in > 2000 CN profiles from the Pan Cancer WGS studies and was able to time SCNA/LOH events relative to the WGD. MEDICC2 also identified mirrored subclonal allelic imbalance and parallel evolutionary events [11]. Taken all together, MEDICC2 applied to high depth NGS data can elucidate early clonal events including WGD along with the subsequent diversity of SCNA.

## Estimating clonal relatedness from somatic mutation data

With the rise of next-generation sequencing, somatic point mutations are increasingly important and offer a high degree of sensitivity for clonality detection. A specific base change is less likely to be shared by chance compared to a large-scale copy number event, and there are often dozens, if not hundreds, of independent mutations to measure in a tumour. Various statistical approaches can be utilised to investigate clonal relatedness in a tumour pair including concordance measures and phylogenetics approaches. The selection of a method may depend on the type of sequencing data available, with phylogenetics-based methods requiring higher depth WES/WGS data.

### Clonality index

Schultheis et al. [7] described and validated a method of estimating clonal relatedness of 23 synchronous endometrioid endometrial carcinoma (EEC) and endometrioid ovarian carcinomas (EOCs) utilising high-depth WES (105×) and targeted sequencing (453×, > 300 genes) (Table 2). They employed two novel approaches for clonality estimation (Clonality Index (CI) 1 and CI2) both based on shared non-synonymous (i.e. mutations likely to alter fitness by changing protein structure and function) and synonymous (i.e. that are likely neutral) somatic mutations. It is noteworthy that they stringently curated the mutation list prior to the CI analysis including manual evaluation in Integrative Genomics Viewer for high confidence variants and filtering out common polymorphisms. The CI takes into account the frequency of a given somatic mutation in TCGA data of the same tumour type. This approach is to reduce the effect of a given mutation in highly frequent driver genes such as hot spot mutations in *PIK3CA* or *KRAS*, which have the potential to mislead the clonality analysis by being shared by chance.

**Table 2:** Summary of the statistical approaches for clonality analysis from bulk DNA sequencing

Author/publication	Technology, type of genomic profile	Tissues (types of tumour), Samples, sample types, treatment types	Method 1	Method 2	Method 3	Preferred method by the author(s)	Significance
Waldman et al. 2000 [4]	CGH arrays, SCNA	FFPE (DCIS), *n*=18, primary DCIS and recurrent/new primary DCIS, BCS w (*n*=3)/wo radiotherapy (*n*=15, *n*=1 unknown); 1 mm surgical margin (*n*=9), positive margin (*n*=6, *n*=3 unknown)	**%concordance** between pairs based on only SCNA (see text for details)	Unsupervised hierarchical clustering based on **pairwise similarities of overall SCNA**	In house developed **similarity score** based on CNA of each chromosome arm with greater weight being given to agreement when a gain or loss was rare than when it was common (see text for details)	None	17/18 were classified as pairs based on Method 1 and 2; 16/18 were classified as pairs based on Method 3; 1 discordant case was common among all methods
Teixeira et al. 2004 [13]	CGH arrays, SCNA	FF (IBC), *n=*12, ipsilateral/bilateral DCIS/LCIS and/IBC/ILC, N/Av	**Probabilistic model** derived by Kuukasjarvi et al. 1997 [40] Probability = dividing number of occurrences of that particular genetic alteration/number of tumours analysed	**Unsupervised hierarchical clustering:** analysis were complete linkage and Pearson correlation: dendrogram was drawn based on overall SCNA or changes in chromosome arm	N/A	None	All cases were concordant between these two methods except one (patient 12 presented with both ipsilateral and bilateral tumours i.e. two in each breast)
Bollet et al. 2008 [6]	SNP arrays, SCNA	FF (IBC), cases *n*=22, control *n*=44, ipsilateral recurrent/new primary IBC, BCS w/wo radiotherapy	Hierarchical clustering of **overall SCNA profile**; Pearson correlation was used to derive a dendrogram	**Shared breakpoints**; M score, partial identity score with a high cut off value (see text for details)	Clinical definition with matched histopathological subtypes, location of the recurrent tumour, grades and hormonal status (see text for details)	Shared breakpoints/partial identity score outperforms the clinical definition/overall SCNA	Method 2 provides significant difference for metastasis free survival than Method 3 (*p*=0.01)
Clonality R Package 2011 [27]	CGH arrays (SCNA, LOH), Mutation analysis (NGS)	Publicly available data as well as own cohort: FF, FFPE: IBC, lobular Carcinoma in situ [5, 26]	SCNA (**chromosome arm as a unit of analysis**), LOH analysis, shared mutations	N/A	N/A	N/A	Statistical approach deriving *P* value for each tumour pair separately for CN and mutations.
Updated Clonality Package 2019-2020 [42]	Mutation analysis (NGS)	As above	Estimated marginal probability of occurrence of a shared mutation in TCGA, Test developed only for metastasised tumours at a different site [43]	N/A	N/A	N/A	Updated R package estimating individual probability for clonal relatedness.
Newburger et al. 2013 [44]	WGS (median 53.4x), somatic SNVs, aneuploidy	FFPE (matched normal, early neoplasia w/wo atypia, carcinoma), *n*=6, synchronous breast early neoplasia with IBC w/wo DCIS	Using **shared somatic mutation**, lineage trees were built. Raw reads were aligned to the UCSC build hg19 and SNVs were called using GATK	N/A	N/A	N/A	IDC had 2.5x private somatic SNVs than the early neoplasia, and 10x than normal tissues; 4/6 cases early neoplasia shared a common ancestor (neoplasia and IDC shared a significant number of SNVs); genome of shared ancestor are already aneuploid.
Weng et al. 2015 [45]	TSP, SNV	FFPE (synchronous early breast neoplasia with IBC and/ or DCIS); *n*=6	Highly accurate **VAF of phylogenetically informative SNVs** was used to build high resolution lineage trees.	N/A	N/A	N/A	Atypical hyperplasia (AH) and DCIS/IBC shared a most common ancestor while DCIS/IDC have more private SNVs than AH. AH also has a greater mutation burden than typical ductal hyperplasia lesions.
Schultheis et al. 2016 [7]	WES (105x n=5), TSP (453x; n=18): method validation b/w WES & TSP (*n*=5); mutational landscape	FFPE (synchronous endometrioid endometrial and endometrioid ovarian carcinoma) *n*=23, metastasis/synchronous/independent primary tumour	**CI**: **based on only nonsynonymous and synonymous mutations** and compared with the frequency in TCGA EEC dataset of a given mutation: ≤20% frequency of any given shared mutation in TCGA is sufficient to call the case to be clonal CI ≥ 0.8	CI2: based on somatic mutations and their frequency in TCGA or a given cohort	N/A	None: CI and CI2 provide concordant results: CI was used by others in multiple studies [8, 46, 47]	CI was discordant with clinical definitions (22/23 was clonal *vs* 15/23 was independent, 8/23 was metastasis, respectively).
Biermann et al. 2018 [12]*	aCGH, SNP arrays (SCNA), DNA methylation, Whole RNA sequencing	FFPE (IBC), *n*=37, independent/new primary tumour (metachronous/synchronous)	-**Similarity Index** (SI) (SCNA) = Shared changes/shared + unique + opposite -**Unsupervised hierarchical clustering** (SCNA, LOH) -**Distance measure**: Euclidean distances	-**Shared Segment analysis**: The breakpoint and CN of each segment was compared between pairs -**Shared mutation**	**Clonality R Package** for SCNA, LOH, mutational data	SI	-Discordance between methods with clinical definition - Clonality analysis by SI are in agreement with other approaches except 5 patients
Roth et al. 2014 [48]	WGS, WES (>100×)	1000 Genomes Project samples	Hierarchical Bayes statistical model: PyClone estimates the clonal architecture and composition using somatic mutant allelic fractions adjusted for sequencing errors, tumour cell content, ploidy and local CN profile	N/A	N/A	N/A	Subclonal reconstruction utilising somatic mutations
Deshwar et al. 2015 [49]	WGS	Simulated data	PhyloWGS: subclonal reconstruction using both somatic mutations and SCNA	N/A	N/A	N/A	Incorporates critical contribution of SCNA for subclonal reconstruction
Kaufmann et al. 2021 [11]	High depth WGS	Pan-cancer Analysis of Whole Genomes, *n*=2778	MEDICC2: defines minimum event distance between pairs of SCNA profiles and uses neighbour-joining to infer relatedness	N/A	N/A	N/A	Incorporates whole genome doubling events

*N/A* not applicable, *WES* whole exome sequencing, *TSP* targeted sequencing panel, *WGS* whole genome sequencing, *SCNA* somatic copy number alterations, *SNV* single nucleotide variants, *VAF* variant allele frequencies, *BCS* breast conserving surgery, *CI* Clonality index, *NGS* next-generation sequencing, *FFPE* formalin-fixed paraffin-embedded, *DCIS* ductal carcinoma in situ, *IBC* invasive breast cancer. *Methods are described only for SCNA, LOH and mutational data. *MEDICC* minimum event distance for intra-tumour copy-number comparisons

The CI for a tumour pair was defined as:

$$ CI=\left\{\begin{array}{c}1-{\Pi}_{k=1}^n{f}_k,n>0\\ {}0,n=0\end{array}\right. $$

where *f*_*k*_ is the percentage of tumours TCGA [50] harbouring a given mutation (*k)* and *n* is the number of shared mutations between a pair of tumours. The pair is considered genetically related if CI is more than 0.8. This approach means that irrespective of the number of shared mutations between a pair of tumours, if just one of the shared mutations has a ≤ 20% frequency in TCGA, then for that pair CI ≥ 0.8 (i.e. clonal).

CI2 is similar to CI, but by using an R package ROCR, the calculated threshold can be carried out with any external large dataset if TCGA data is not available for a particular tumour type. Unlike CI, where the threshold was fixed as 0.8 to define any pair tumour as “clonal”, the CI2 threshold will vary based on the average mutation rate of that tumour type in the control dataset, and the frequency distribution of variants of the testing cohort (calculated by the ROCR package).

Very recently we employed this analysis in 8 breast papillary lesions with co-existing DCIS/IBC using targeted sequencing. Despite a very low mutation burden (median of one shared mutation/case) 6 cases were found to be clonally related by the CI index, driven by shared variants infrequent in breast TCGA data [18] and consistent with clonality determined by copy number.

The risk in this CI analysis is that if the particular tumour type (or subtype) has a common hot-spot driver mutation along with a low mutational burden and/or employment of a targeted panel, estimating clonal relatedness could result in equivocal findings. In addition, the mutational landscape markedly differs among subtypes of breast cancer reported in TCGA. For example, the most commonly mutated gene in breast cancer is *PIK3CA* (36%), however, when we look at the frequency of different subtypes, *PIK3CA* mutation is found in 37% of luminal subtypes/ER+ tumours (45% in Luminal A, 29% in Luminal B) *vs* 9% of basal/ER- tumours. But how far should the reference set go in terms of subtypes? For example, even within the breast cancer luminal subtype there is further heterogeneity in terms of gene expression and histological subtypes (i.e. ductal/lobular/mucinous/papillary); should these be treated separately? Comparing mucinous IBC with luminal IBC from both TCGA and METABRIC showed a different mutational profile, namely for *PIK3CA* (6.7% mucinous *vs* 37.4% IBC of no special type, *p*< 0.001, Fisher’s exact test) and *TP53* mutation (3.3% *vs* 20%, *p*< 0.02, Fisher’s exact test) [51].

Another major concern common to any mutation-based approach is that they rely on accurately identifying somatic variants. Should an uncommon germline polymorphism be present that is wrongly classified as somatic, this will of course be present in both tumours and they will be called as clonal (i.e. one rare germline/sequencing artifactual mutation can significantly define CI> 0.9). Therefore, matching germline data and stringent variant filtering are essential.

### Statistical tests for relatedness of a tumour pair

Statistical tests, similar to those described earlier for SCNA and LOH can also be applied to single nucleotide variant data, using mutation frequency from TCGA or an independent external dataset for subtypes of a cancer type (e.g. Luminal A breast cancer) [41]. For example, the *p* value generated by the Clonality package “*get.mutation.frequencies*” function is based on how common the shared variants of the pair in TCGA dataset. The less common it is, the smaller the *p* value will be and therefore, suggestive of a clonal pair if *p* < 0.05. This package also has a random-effects model function to estimate independent probabilities of clonal relatedness based on unshared mutations between paired tumours when the second pair is at the same site (i.e. not metastasis). Mathematically, Mauguen et al. showed that with increasing unshared events, the probability of a pair being clonal also decreases [42]. This test cannot provide the probability of independence in cases with no matches (i.e. *p* value is always 1, regardless of how many non-matches occur in the case).

### Subclonal reconstruction with high depth bulk sequencing data

Better understanding of ITH is important for future biomarker discovery as well as therapeutic interventions. In order to understand the heterogeneity in a single tumour, subclonal reconstruction is a necessary step to distinguish between clonal and subclonal populations of the tumour. The major challenges of bulk DNA sequencing analyses have been described in detail by Tarabichi and colleagues [52] and include the type of sequencing, sequencing coverage, single *vs* multiple sampling to reconstruct subclones, tumour ploidy, purity, and germ line variants being misclassified as somatic.

One widely used computational tool for subclonal reconstruction is PyClone, which can be applied to multi-sample analyses as well as to a single tumour. PyClone estimates the clonal architecture and composition by using mutant allelic fractions from all somatic mutations adjusted for sequencing errors, tumour cell content, ploidy and local CN profile. However, PyClone assumes only one chromosomal change per segment. Generally, PyClone is recommended for samples with high depth (e.g. at least 100× sequencing depth for WES) and with matched normal data [48, 52]. Lower depth of matched normal might overestimate clonal mutations through the misclassification of germline variants as somatic (i.e. false positive clonal relationship). Additionally, generally deeper sequencing improves the accuracy of subclonal reconstruction. For calling clonal relatedness of paired samples, a tree with no shared trunk would indicate lack of clonality, as might a very short trunk defined by a highly frequent mutation potentially shared by chance. PyClone was utilised on high-depth WES data to describe synchronous DCIS progression to IBC using single-sampled specimens [53] and for multi-region sampling in the TRACERx-NSCLC cohort to define clonal *vs* subclonal mutations as well as the timing the events (early *vs* late) [22].

### Evolutionary methods with high depth bulk sequencing data

PyClone only utilises somatic point mutations to reconstruct subclonal populations and ignores the critical contribution of CN changes. In contrast, PhyloWGS combines both types of event and predicts more accurate subclonal reconstruction than other tools since it recognises the impact of CN changes on variant allele frequencies (VAF). For example, both CN amplification and LOH can cause increased VAF but would be indicative of a separate subclonal lineage. An increased VAF might therefore be ambiguous if the CN data is not incorporated [49]. As for PyClone, PhyloWGS could also be used to estimate the clonal relatedness of two tumour samples.

Taken together, in order to identify whether a tumour pair (e.g. primary/recurrence or primary/metastasis) share a common ancestor, the clonality index and clonality packages are widely used with data from targeted sequencing panels and WES. If higher depth WES or WGS data are available, phylogenetic quantification would be most powerful for both single-sample subclonal reconstruction and multi-sample clonal relatedness analyses.

## Consideration of biological contexts when determining clonal relatedness

When analysing higher depth NGS data, consideration of biological contexts might not be so crucial, since >60× coverage will precisely define breakpoints, provide allele-specific CN changes and estimate evolutionary distance. However, before any of the discussed methods (Table 2) are applied, biological contexts should be taken into account, especially when working with low resolution technologies.

### Initiating events for cancer evolution, premalignant lesions and estimating clonal relatedness with synchronous cancer

It has long been understood that cancer-initiating events are often tissue specific, such as *APC* mutation in colon carcinoma, *DNMT3A* mutation in AML, and loss of chromosome 16q in breast cancer [54]. Understanding and unravelling these initiating events, how they increase fitness and are clonally selected, gradually expand and progress further to cancer, might open windows for prognostic tools or preventative therapies. Here in this Review, “premalignant” lesions will be referred as clinically diagnosed lesions which may be considered as a precursor to malignancy, summarised by Curtius et al. [55].

Premalignant lesions most likely will have a different clonal architecture than fully developed cancer. Therefore, to analyse premalignant lesions and their clonal relatedness with synchronous/metachronous cancer, it is important to remember some key points:

1. Generally, the degree of aneuploidy is higher with the progression of cancer with the exception of breast and lung cancer [56], whereby the in situ diseases of both types have similar CIN to invasive tumours [56]. In addition, the pattern of mutations and aneuploidy is known to be tumour-type specific [57].
2. Although there is growing understanding of the somatic mutational landscape in phenotypically normal tissues [58, 59], such as endometrium [60], skin [61] and oesophagus [62]; a higher degree of somatic mutations are still observed in cancer than normal tissues. This observation indicates that only a few of the many driver mutations of each tumour type already exist in normal tissues and could be sufficient to drive clonal expansion, albeit often still behaving phenotypically as normal tissue. Therefore, it is possible that some driver mutations, if not all, are expected to be more frequent in premalignant conditions than the passenger mutations of a malignant tumour (i.e. that passively accumulate with the progression of the disease) [55]. For example, it was shown in melanoma that only one fully clonal mutation (mostly *BRAF* V600E) was present in both the benign precursor and coexisting invasive melanoma, with an increased rate of other somatic mutations after disease progression through intermediate lesions [63].
3. Observed premalignant lesions may or may not be a direct precursor to a coexisting or later cancer, therefore, while the two neoplasms may share a very early common ancestral cell, premalignant lesions could follow an early divergent evolutionary trajectory. This early branching evolution may manifest as significantly fewer genetic alterations overall in premalignant lesions than malignant tumours. Such branching evolution was shown by phylogenetic tree analysis of somatic SNVs by Weng et al. and others [44, 45] for early breast lesions and DCIS/IBC (*n*=6).

While all of the statistical approaches described in this Review so far have been tested on genetically advanced in situ lesions or invasive cancers in recurrence/metastasis settings, a question remains: which method should be employed in pre-malignant settings? For example, for breast premalignant lesions, such as ADH synchronous with IBC/DCIS, even if ADH is a direct precursor to cancer, it is still expected to have fewer genetic alterations than the DCIS/IBC counterparts. More importantly, as mentioned above, some ADH cases might carry only the initiating genetic events/driver events (i.e. the most frequent changes in DCIS/IBC), such as whole chromosome arm 1q gain and/or 16q loss (~ 60% and ~ 80% of breast cancers carry these changes, respectively). The genetic relationship of ADH to DCIS/IBC depends on the relative duration of the period in which the original clonal cell accumulated those initiating events and became “ADH” compared to the period of ADH and the DCIS evolving separately. If that relative duration is very small, then only initiating events are expected in ADH lesions compared to co-existing carcinoma. This diversion will be especially wide with high-grade DCIS or IBC, whereby the carcinoma component will likely evolve on its own with a high level of genomic instability. In addition, multifocal ADH might be heterogeneous with regards to their CN events but due to the limitations of bulk sequencing, we cannot detect such spatial heterogeneity. Only one clone might eventually give rise to cancer and only the clones that are histologically “ADH”, while still sharing the common ancestor, are likely to be analysed with bulk DNA sequencing.

Approaches that take the frequency of genetic events into account for clonality assessment, such as the Clonality Package, will have limited utility, since they discount the common changes of the dataset being tested. For example, one of the cases in the study by Begg et al. [5] provides an example of how the impact of frequent and shared SCNA is reduced. In this case, only 1q whole arm gain and 16q whole arm loss were shared between lobular carcinoma in situ (LCIS) and invasive lobular carcinoma (ILC); this case was called as non-clonal due to the high frequency of these events in the dataset (*P*=0.31).

We recently showed that ADH is a multipotent precursor of IBC using only CN data [64] and employing unsupervised hierarchical clustering, the Clonality Package and visually inspected shared breakpoints. The discordant cases among these three methods arose primarily due to the lesions only sharing CN changes very frequent in IBC. Some were defined as clonal based on the precise shared breakpoints of those common events. Additionally, it is important to note that premalignant lesions of a heterogenous tumour type such as breast need to be classified on their own first (without synchronous cancer) in order to anticipate the frequency of any genetic events in those lesions when they exist by themselves. For example, isolated breast papillary lesions have almost no CN events [18, 64] and therefore, might share only one or two common CN events with the DCIS/IBC, again depending on the evolutionary context of that particular case.

The ability to interrogate premalignant lesions has been technically challenging, due to their small size and their low nucleic acid quality due to fixation with formalin. Nonetheless, Hu et al. [65] recently investigated lung pre-neoplasia to understand the progression to lung adenocarcinoma whereby cases were subjected to multi-region WES (at least 20×) to estimate clonality and phylogenetic analysis. Ideally, single cell sequencing or higher depth sequencing using WES/WGS would be performed whereby early divergence and the duration of branching evolution would be taken into account for more accurate clonality estimation. Lacking data with such high-depth and breadth, a new statistical approach or test is necessary to accurately evaluate cancer progression from premalignant lesions synchronous or metachronous with cancer. An approach that incorporated specific events (breakpoints, mutations) and incorporating their frequency in a relevant control tumour set could be the most suitable.

### Detecting clonality in metastasis

When determining the clonality of tumours at anatomically distant sites, which reference cohort should be used? Mutational profiles, especially the driver events, differ substantially based on the site of the origin. If there are very few shared mutations how will the marginal probabilities be calculated? And even if they are defined as clonal, which site will be considered as the primary in a synchronous context? The importance of biological context has very recently been addressed in an extension of the Clonality package [43]. The updated equation for different anatomical locations now assumes rather than a shared probability for both samples, that the different cell types have different mutation probability distributions. A classic example was given: two tumours were subjected to targeted sequencing using a panel of 410 genes, one located in the pancreas (*n*=8 mutations) and one in lung (*n*=9 mutations), whereby they shared only one mutation, *KRAS* G12C. The original test derived a non-significant *P* value for this case (*P*=0.076), however, the updated test calculating the “incongruence index” (i.e. the index that estimates the likelihood of each site being the primary site) was suggestive of a lung cancer metastasized to the pancreas. This result is due to the much lower abundance of the only shared mutation in the pancreas compared to the lung (0.8% *vs* 12.8%, respectively), meaning the tumour was unlikely to have arisen in the pancreas independently of the lung tumour. This example highlights the importance of taking into account the biological context (which in this example is tumours at anatomically distant sites) before employing any statistical approaches.

It is important to remember a few key points with regards to metastatic settings in utilising statistical tools for determining clonality:

1. Evidence of early dissemination for distant metastasis, even before primary tumours become clinically apparent, has been identified for multiple solid tumour types [66]. This process implies that only truncal mutations will be shared between primary and distant metastases and functional driver mutations will be enriched on the trunk of the phylogenetic tree [66]. Therefore, approaches that also consider differences as well as similarities (e.g. hierarchical clustering) may not be ideal for detecting clonality in this scenario.
2. Similarly, multi-region sequencing suggested that in colorectal cancer, distant metastases arose only from a small region of the primary tumour [67], thus if that region is not sampled for analysis, then only truncal mutation(s) will be reported as shared. The clonality estimation will depend on how well the tumour was sampled—bulk DNA analyses could be underpowered to detect sub-clonal mutations (depending on the sequencing coverage). While truncal mutations will still be detected in both sites, detection of mutations at a low frequency in the primary and enriched in the metastatic site could increase the power of clonality detection, which is especially important if there are no clear truncal mutations or a truncal mutation is a common variant.
3. Highly divergent CN profiles are common in metastatic tumours compared to the primary, and the nature of chromosome instability is that later events build on and can obscure initiating breakpoints (e.g. in the context of breakage-fusion-bridge cycles [68]).
4. Reiter et al. [67] suggested that genetic diversity might be different in local lymph node metastases compared to distant ones, whereby a very tight selective bottleneck and therefore less genetically diverse clones might be expected in the latter. Similarly, Hu et al. [66] evaluated colorectal, breast and lung tumours (*n*=136 by WES) and found that monoclonal tumour deposits (i.e. originating from a single cell or clone) are more frequent in distant metastasis than the primary. In contrast, for other tumour types, such as ovarian cancer, polyclonal seeding is not uncommon [69, 70]. The prevalence of different metastatic seeding events is important to keep in mind if we would like to estimate clonality in metastasis settings.
5. Treatment influences clonal evolution and the mutation landscape in metastatic settings. For example, higher mutational burden in metastases than the treatment naïve pancreatic ductal adenocarcinoma [71]. In addition, the frequency of specific mutations may be higher, such as mutations in *ESR1* in breast cancer metastasis after anti-oestrogen therapy [72]. These features should be considered when applying a burden or frequency-weighted approach.

While the evolutionary trajectory of distant metastasis is yet to be explored with large single-cell-sequencing studies across multiple tumour types, it is important to take the context and clinical information into account while employing statistical clonality tools.

## Conclusion

With the growing body of evidence in the field of cancer evolution and the importance of tumour heterogeneity to clinical outcome, biological context is increasingly important. Since high depth, breadth and single cell sequencing technologies are becoming more and more feasible, clonal analysis is an important tool to unravel the biology of the heterogeneous populations of any tumour type. For example, it has long been an ongoing challenge to identify predictive biomarkers for DCIS recurrence, especially when they recur as IDC. Unless single cell analysis or multi-region high depth WES/WGS analysis is carried out, the genomic complexity of the primary tumour might be severely underestimated, and the genetic relationship between the primary and recurrence misinterpreted. There might be subclonal events, not well represented in the specimen sequenced, carrying any of the genetic events that have the potential to drive DCIS progression towards invasiveness over time. In addition, it is an active field of research to understand how in breast cancer, immune “cold” regions co-exist with immune “hot” regions and whether the prognosis is different for patients with such potentially genetically determined heterogeneity. Without unravelling the intra-tumoural spatial heterogeneity, we might not be able to determine the evolutionary bottleneck of DCIS progressing to IDC. With greater depth, we can also estimate the selection type (positive/negative/neutral), which is often impossible due to sampling bias. Additionally, with the potential for further development of single cell genomics technology for FFPE samples, bioinformatics tools also should be developed to overcome the caveats of current analytical tools. Single cell analysis may provide deep insights into tumour evolution and the order of the genetic events, such as the punctuated model for triple negative breast cancer [10].

Most of these tools require matched normal data, yet the feasibility of obtaining germ-line DNA is problematic for retrospective studies using archival tissue samples with long follow up or those collected through routine diagnostic procedures. Another point to consider is that normal tissues have been shown to carry potential driver mutations [59, 73] and can be aneuploid (e.g. normal colorectal crypts [74] and non-malignant cells like fibroblasts and immune cells [75]). Should the pipeline be modified when we analyse the variants of cases with matched normal, particularly when single-cell approaches are employed? Other limitations of clonality analysis include the inherent noise of some assays such as FFPE-CN profiling, which then require manual inspection. Advancement in methods to reduce noise without manual curation will dramatically improve the accuracy and ease of analysis.

Bulk sequencing is still an important ongoing tool given the well-established pipelines in a wide range of tissue sources [76], but lineage tracing and single cell technologies have shown great promise to study cancer evolution. Exciting developments in spatial single-cell technologies have the potential to one day evaluate entire cancer tissue fields for their clonal relationships in the context of the microenvironment and immune escape mechanism. All of these developments to estimate clonal relatedness might have the potential to aid in clinical treatment strategies in the future.

## Supplementary Information

**Additional file 1.** Review history
