# Supplementary material for: Evaluating statistical approaches to define clonal origin of tumours using bulk DNA sequencing: context is everything
Source: Genome Biol. 2022 Feb 2;23:43. doi: 10.1186/s13059-022-02600-6 (PMC8809045; doi:10.1186/s13059-022-02600-6)
Supplement: Supplementary file 1 — Additional file 1. Review history [file 13059_2022_2600_MOESM1_ESM.docx]

Review History

**First round of review**

**Reviewer 1**

**Comments to author:**

The authors present a review of approaches for inferring the clonal relatedness of cancer samples. Their focus is on approaches using bulk DNA omics technologies including array and NGS based approaches. To highlight real world applications the author's have included vignettes from breast cancer studies, their area of expertise.

The manuscript is well written and generally easy to follow. The review seems to cover most relevant methods though with a skew towards older technologies and approaches. While I feel this manuscript would be a good fit for a more specialized cancer focused venue, I do not see that it would be of interest to a broad readership such as Genome Biology. Below I outline points for improvement.

Major comments:

The topic matter seems relatively narrow and primarily focused on identifying whether patient samples are clonally related. This seems like a narrow application and doesn't capture many of the other interesting applications of clonal analysis that have occurred in recent years.

The methodology discussed is quite old, particularly the array based methods. While perhaps clinically interesting still, much of the work in this area was well established a decade ago. I was surprised by how much emphasis has been placed on total copy number based approaches as opposed to more refined allele specific copy number profiles. Next generation sequencing is covered to a lesser extent. Single cell sequencing is only briefly mentioned.

To some extent the manuscript felt more like a walk through of the features of the Clonality package as opposed to a general review of the field.

The discussion of future directions is very limited, largely relegated to a single paragraph in the discussion. I would be much more excited to see details on how these new approaches are beginning to be applied to study clonality and what potential benefits/pitfalls they have versus the long established approaches discussed in the manuscript.

I think the emphasis on context, both in terms of disease type and platform, was well done. The manuscript would benefit from a table or figure that highlights the different platforms and their benefits/deficiencies.

A schematic figure illustrating the different indexes computed would be helpful i.e. what cases would lead to high clonality between samples vs low.

Minor comments:

Some of the formulas are difficult to follow due to formatting. Adding parenthesis around the denominator of ratios would help. For example the definition of the SI index.

The description of triple negative breast cancer heterogeneity is confusing. In one sentence low inter-tumour heterogeneity is ascribed to TNBC but then the next one details how single cell sequencing has shown heterogeneity.

The assertion SNP arrays provide more precise breakpoints than NGS needs to be clarified. Presumably NGS sequenced to the standard 60-100x would be vastly more accurate for breakpoint definition than SNP arrays.

The manuscript does not end up providing clear guidance on the best approaches in the pre-malignant setting.

Some technical ideas like "waviness of the ratios" is not adequately explained for a general audience.

**Reviewer 2**

**Comments to author:**

In their review article "Evaluating statistical approaches to define clonal origin of tumours using bulk DNA sequencing: Context is everything" the authors provide a literature review of a number of methods designed to determine "clonal relatedness" or "clonality" between tumour genomes analysed with whole-genome techniques including sequencing and microarrays.

The review tackles an interesting, timely and highly relevant area of cancer genomics, which can be difficult to get into for new researchers to the field and the review provides in-depth description of several interesting papers from the field. I liked the level of detail with which some methods were described and thought it courageous to tackle such a wide array of different methodologies together with their experimental and data context.

However, I also had some major issues with the manuscript in its current form which I will try to outline here. As a disclaimer, my expertise is in methods for inferring cancer evolution from SCNA data and their clinical applications.

Firstly, I had to read the manuscript several times to actually get a grasp on what the precise topic of the review is. The problem is that "clonal relatedness" and "clonality" are not well-defined terms in cancer genomics and this is reflected in this article. The authors stray from general pairwise comparisons between genomes to concrete questions, such as monoclonal vs polyclonal seeding of tumours (a controversial topic in and of itself). Ultimately, the authors review (mostly) pairwise similarity or dissimilarity measures between whole genomes or parts thereof that are based on mostly SCNA (and some on SNV) readouts, but intermingle these summary statistics with discussions of different clustering algorithms (hierarchical clustering) or different ways of segmenting SCNA data (circular binary segmentation). The manuscript would benefit here from a more well-defined topic, for example clearly state that the authors are interested in reviewing pairwise similarity measures for estimating the degree of clonal relatedness, but not go into details about e.g. segmentation algorithms or the like.

Along the same lines, I also found the structure of the review itself hard to grasp with multiple different levels of heading and subheadings, some with, some without consecutive numberings (a, b, etc.), some underlined, some not, which made it very hard to follow the overall train of thought. The manuscript would benefit strongly from a much simplified structure following a precise definition of the scope of this review.

Here, it must be pointed out, that estimating the degree of relatedness between a pair of tumours ultimately requires an evolutionary model and most of the presented methods are simple summary statistics, which, while certainly useful, all are somewhat ad-hoc measures that were created because no sufficiently complex evolutionary model exists. Here, the field is clearly moving towards methods with an explicit evolutionary model, such as PyClone or also MEDICC, which the authors cover, albeit not in sufficient detail. For example, MEDICC combines breakpoint and segment-based SCNA analysis by defining a minimum-event distance between a pair of genomes. I would strongly suggest to expand this paragraph, and also define the measures defined in these papers and reference the latest versions (e.g. https://www.biorxiv.org/content/10.1101/2021.02.28.433227v1). It should also be made clear in the review, that ultimately, evolutionary models will be required to certainly answer questions about clonal relatedness.

A clear line further needs to be drawn between the similarity or dissimilarity measure (e.g. similarity index or minimum event distance) and the method designed to infer the clonal hierarchy or phylogenetic relationship between the samples or clones (e.g. hierarchical clustering or neighbour joining). Again, here, as the authors correctly state on p.19, hierarchical clustering is generally a poor choice, as here "differences as well as similarities [...] may not be ideal for detecting clonality". Here, also, evolutionary methods, such as neighbour joining, or minimum evolution (https://pubmed.ncbi.nlm.nih.gov/14694080/) have been shown to be advantageous.

The necessity for an evolutionary model also becomes clear in the authors' discussion about how "independent tumours are quite likely to share CNA at commonly affected loci by chance" (p.7). This does not only affect independent tumours. Parallel and convergent evolution has been demonstrated to be ubiquitous across human cancers (https://pubmed.ncbi.nlm.nih.gov/32879494) (https://pubmed.ncbi.nlm.nih.gov/28445112) and will mislead simple statistical approaches that assume that shared events imply similarity by descent. This issue is even maintained in many evolutionary models which make use of the infinite sites assumption.

In summary, the review would benefit from restructuring and a clear definition of what its scope is. The authors must reference the evolutionary context more clearly. In addition, some of the "context-specific considerations" are really basic problems of dealing with a certain type of data. For example, point 4) that SCNA events "build on and can obscure initiating breakpoints" (p.20) is solved by event-based evolutionary approaches such as MEDICC, which the authors briefly discuss.

As a side-note, with a heavy focus on SCNA analysis, the review does not mention whole-genome doubling events as a key indicator of clonal relatedness, which I consider an oversight and should be addressed. Also, there is no mention of allele-specific vs total copy-number, an important distinction between aCGH data and NGS or SNP array data which enables inclusion of LOH events and parallel evolution and might be useful to mention in the paragraph about LOH events.

Minor points

The abstract is very short and would benefit from a clearer description of what this review covers

p.3: the authors state that "Various technologies were employed such as [...] CGH arrays or SNP arrays. In this review we will focus on more advanced technologies [...] (i.e. CGH arrays, SNP arrays)". I assume the first sentence was supposed to name less advanced techniques? Then I would remove the reference to CGH and SNP arrays from the first sentence.

p.4 - l37: "considered as an event": It is unclear what an event is here, this needs to be defined.

p.5 - l.57: "Thus, clustering together of two tumours from the same patient readily indicates the clonal relatedness": Really the only thing it indicates is that they are similar according to the statistics employed. This similarity might or might not have to do with relatedness (see comment above about evolutionary model, parallel evolution etc.).

p.5 - l.61: "(percentage concordance, hierarchical clustering, similarity score)": These are not alternative methods. Percentage and similarity score are both similarity scores, whereas hierarchical clustering is a clustering method which also requires a distance (dissimilarity) measure, typically euclidean (which is also non-evolutionary). Supposedly, concordance and similarity scores were also subjected to some clustering in this study.

p.6 - l.75-80: "Given this high frequency of chromosomal changes, it is possible that many pairs would show clonal relatedness by chance.": they might show similarity by chance which erroneously would be interpreted as clonal relatedness. This is not necessarily a problem of SCNAs as such, but of the method used to infer relatedness (event-based methods are likely to do much better).

p.6 - l.83: Please reference a suitable test dataset if you have one in mind.

p.10 - l173: " (i.e. SNP arrays provide more precise breakpoints than NGS).": That's not true, please remove. Generally NGS data has improved resolution and precision over SNP arrays, and nucleotide-level breakpoints via split-read analysis cannot even be performed with SNP arrays.

p.11: The excursion into SCNA segmentation (CBS etc.) seems superfluous.

p.17 - l.354: Pleas also cite https://pubmed.ncbi.nlm.nih.gov/25293804/

p.17 - l.362: PyClone does not only take SCNA input from ABSOLUTE or FACETS. I would remove the specific SCNA callers or alternatively be complete and add at least ASCAT and SEQUENZA and add references for all.

p.18 - l.370: Please refer to the latest version and provide the minimum event distance ( https://www.biorxiv.org/content/10.1101/2021.02.28.433227v1)

p.25 - l.543: I would not agree, in most clinical studies blood is taken as a control tissue, which is fully sufficient for SCNA analysis, does not typically contain many somatic mutations. Further, normal tissues are rarely "enriched with aneuploidies" (depending on the tissue), this is actually a major difference to SNVs (of which they have plenty).

Figure 1: I don't think I fully understand the Figure, many of the discussed similarity scores are independent of the underlying technology. In particular SNP arrays and WES/WGS are often times exchangeable for SCNA analysis. The figure would also benefit from some visual improvements including a cleaner layout and less text.

**Authors Response**

**Point-by-point responses to the reviewers’ comments:**

*We thank both reviewers for their valuable suggestions and constructive feedback to improve the submitted MS. Major changes are highlighted in yellow in the revised MS. In addition to the requested changes, we have had the paper edited for readability and brevity by a colleague.*

REVIEWER #1:

"The authors present a review of approaches for inferring the clonal relatedness of cancer samples. Their focus is on approaches using bulk DNA omics technologies including array and NGS based approaches. To highlight real world applications the authors have included vignettes from breast cancer studies, their area of expertise.

The manuscript is well written and generally easy to follow. The review seems to cover most relevant methods though with a skew towards older technologies and approaches. While I feel this manuscript would be a good fit for a more specialized cancer focused venue, I do not see that it would be of interest to a broad readership such as Genome Biology. Below I outline points for improvement.

Major comments:

The topic matter seems relatively narrow and primarily focused on identifying whether patient samples are clonally related. This seems like a narrow application and doesn't capture many of the other interesting applications of clonal analysis that have occurred in recent years.

The methodology discussed is quite old, particularly the array based methods. While perhaps clinically interesting still, much of the work in this area was well established a decade ago. I was surprised by how much emphasis has been placed on total copy number based approaches as opposed to more refined allele specific copy number profiles. Next generation sequencing is covered to a lesser extent. Single cell sequencing is only briefly mentioned.

To some extent the manuscript felt more like a walk through of the features of the Clonality package as opposed to a general review of the field."

*We agree with the reviewer that our scope of the initially submitted MS was somewhat limited to older technologies or lower depth NGS data. We have streamlined the section on older technologies, although we argue that these retain some value since in this review since many studies using FFPE material on small lesions are still restricted to low-coverage WGS and panel sequencing. Since both reviewers found that including approaches for NGS data would be an improvement, we have added several paragraphs on recent advancements from multiple large cohort studies, such as TRACERx and ICGC Pan Cancer WGS studies. PyClone and MEDICC have been covered in more detail and we have added a section on how phylogenetic reconstruction of tumours including whole genome doubling has revolutionised our understanding of cancer evolution. We also have added a paragraph about allele-specific CN.*

*All of these changes are highlighted in the MS (Page 5-6 Line 59-69; Page 9 Line 169-178, Page 19 Line 400-408, 412-424, Page 20-21 Line 430-443, Page 21 Line 447-457, Page 22 Line 465-483).*

*In line with the suggestion to make the MS more appealing to a wider readership, we have revised Figure 1 and added a new Table 1 describing the different platforms and their resolution. We also have added new Figures: Fig 2 explains different evolutionary models and Fig 3 explains allele specific copy number changes by high depth sequencing data.*

"The discussion of future directions is very limited, largely relegated to a single paragraph in the discussion. I would be much more excited to see details on how these new approaches are beginning to be applied to study clonality and what potential benefits/pitfalls they have versus the long established approaches discussed in the manuscript."

*We have added additional points to the Future directions section, and provided an example of a clinical problem and how these tools and analysis can help to solve the issue. We have briefly discussed single cell sequencing. Page 23 Line 489-504.*

"I think the emphasis on context, both in terms of disease type and platform, was well done. The manuscript would benefit from a table or figure that highlights the different platforms and their benefits/deficiencies."

*We have added Table 1 describing the different platforms and their utility, as well as a new Figure 1 which better illustrates the types of genetic events detected by these methodologies and how the various tools can be applied.*

"A schematic figure illustrating the different indexes computed would be helpful i.e. what cases would lead to high clonality between samples vs low."

*The new Figure 1 illustrates the advantages and disadvantages of each of the indices and under what circumstances they can be applied.*

"Minor comments:

Some of the formulas are difficult to follow due to formatting. Adding parenthesis around the denominator of ratios would help. For example the definition of the SI index."

*Parentheses have been added to the SI index definition. Some other formulas have been removed.*

"The description of triple negative breast cancer heterogeneity is confusing. In one sentence low inter-tumour heterogeneity is ascribed to TNBC but then the next one details how single cell sequencing has shown heterogeneity."

*We have removed this portion to be in line with other suggestions by both reviewers.*

"The assertion SNP arrays provide more precise breakpoints than NGS needs to be clarified. Presumably NGS sequenced to the standard 60-100x would be vastly more accurate for breakpoint definition than SNP arrays."

*We also have removed this part at the request of other reviewers and we agree that >60x NGS would provide stronger evidence of breakpoints than SNP arrays. Initially this comment was meant for low depth sequencing and hence it created confusion.*

"The manuscript does not end up providing clear guidance on the best approaches in the pre-malignant setting."

*It is a difficult to comment on about the best approaches, as we haven’t yet come across a perfect one for this scenario. However, we have stated the following in the context section (Page 16 line 329-335.)*

*“"Ideally, single cell sequencing or higher depth sequencing using WES/WGS would be performed whereby early divergence and the duration of branching evolution would be taken into account for more accurate clonality estimation. Lacking data with such high-depth and breadth, a new statistical approach or test is necessary to accurately evaluate cancer progression from premalignant lesions synchronous or metachronous with cancer. An approach that incorporated specific events (breakpoints, mutations) and their frequency in a relevant control tumour set would likely be be the most suitable.”"*

"Some technical ideas like "waviness of the ratios" is not adequately explained for a general audience."

*We have removed such details of the lower resolution methodologies to have the space for other changes more relevant to the wider audience.*

REVIEWER #2:

"In their review article "Evaluating statistical approaches to define clonal origin of tumours using bulk DNA sequencing: Context is everything" the authors provide a literature review of a number of methods designed to determine "clonal relatedness" or "clonality" between tumour genomes analysed with whole-genome techniques including sequencing and microarrays.

The review tackles an interesting, timely and highly relevant area of cancer genomics, which can be difficult to get into for new researchers to the field and the review provides in-depth description of several interesting papers from the field. I liked the level of detail with which some methods were described and thought it courageous to tackle such a wide array of different methodologies together with their experimental and data context. However, I also had some major issues with the manuscript in its current form which I will try to outline here. As a disclaimer, my expertise is in methods for inferring cancer evolution from SCNA data and their clinical applications."

*We appreciate the recognition of our effort for this review. We also highly appreciate the level of details provided by the reviewer to make this MS better and more informative.*

"Firstly, I had to read the manuscript several times to actually get a grasp on what the precise topic of the review is. The problem is that "clonal relatedness" and "clonality" are not well-defined terms in cancer genomics and this is reflected in this article. The authors stray from general pairwise comparisons between genomes to concrete questions, such as monoclonal vs polyclonal seeding of tumours (a controversial topic in and of itself). Ultimately, the authors review (mostly) pairwise similarity or dissimilarity measures between whole genomes or parts thereof that are based on mostly SCNA (and some on SNV) readouts, but intermingle these summary statistics with discussions of different clustering algorithms (hierarchical clustering) or different ways of segmenting SCNA data (circular binary segmentation). The manuscript would benefit here from a more well-defined topic, for example clearly state that the authors are interested in reviewing pairwise similarity measures for estimating the degree of clonal relatedness, but not go into details about e.g. segmentation algorithms or the like. Along the same lines, I also found the structure of the review itself hard to grasp with multiple different levels of heading and subheadings, some with, some without consecutive numberings (a, b, etc.), some underlined, some not, which made it very hard to follow the overall train of thought. The manuscript would benefit strongly from a much simplified structure following a precise definition of the scope of this review."

*We have better defined the scope of the review in the abstract and introduction. We have streamlined the section on older technologies and methods, cutting down on unnecessary details. While retaining our review of some of these older and simpler analytical tools, we have added findings from recent publications with higher depth multiregional sequencing data to show how these simpler tools can underestimate complexity of tumour evolution.*

*We have simplified the structure to the following headings and subheadings:*

*Introduction:*

*Estimating clonality utilising copy number events from low depth NGS data or array-based technologies:*

*a) Use of only total SCNA concordance to derive similarity profile*

*b) Estimating clonality based on shared breakpoints*

*c) Estimating clonality based on both SCNA and loss of heterozygosity (LOH)*

*Statistical approaches for the assessment of clonal relatedness based on somatic mutations from bulk DNA sequencing:*

*a) Clonality Index*

*b) Clonality package for mutational profile*

*Context-specific considerations in determining clonality while utlising low depth single sampling NGS or array based technologies:*

*a) Initiating events for cancer evolution, premalignant lesions and estimating clonal relatedness with synchronous cancer:*

*b) Detecting clonality in metastasis*

*Estimating clonality by subclonal reconstruction with high depth bulk sequencing data:*

*a) MEDICC and MEDICC2*

*b) PyClone*

*c) PhyloWGS*

*d) Overall importance of phylogeny reconstruction*

*Conclusion:*

"Here, it must be pointed out, that estimating the degree of relatedness between a pair of tumours ultimately requires an evolutionary model and most of the presented methods are simple summary statistics, which, while certainly useful, all are somewhat ad-hoc measures that were created because no sufficiently complex evolutionary model exists. Here, the field is clearly moving towards methods with an explicit evolutionary model, such as PyClone or also MEDICC, which the authors cover, albeit not in sufficient detail. For example, MEDICC combines breakpoint and segment-based SCNA analysis by defining a minimum-event distance between a pair of genomes. I would strongly suggest to expand this paragraph, and also define the measures defined in these papers and reference the latest versions. It should also be made clear in the review, that ultimately, evolutionary models will be required to certainly answer questions about clonal relatedness.

A clear line further needs to be drawn between the similarity or dissimilarity measure (e.g. similarity index or minimum event distance) and the method designed to infer the clonal hierarchy or phylogenetic relationship between the samples or clones (e.g. hierarchical clustering or neighbour joining). Again, here, as the authors correctly state on p.19, hierarchical clustering is generally a poor choice, as here "differences as well as similarities [...] may not be ideal for detecting clonality". Here, also, evolutionary methods, such as neighbour joining, or minimum evolution have been shown to be advantageous."

*We agree on the direction that the field is taking, however we would argue that providing some detail on summary statistics retains value in this review since many studies using FFPE material on small lesions are still restricted to low-coverage WGS or panel sequencing.*

*We have expanded the sections on PyClone and MEDICC and added information on MEDICC2. We have described findings using both PyClone and MEDICC as to why evolutionary models are important. We also have added a paragraph about WGD events and how MEDICC2 can dramatically improve our understanding by incorporating WGD with SCNA analysis. We have described and illustrated in Figure 2 and reemphasize in the Conclusion why evolutionary models will be required to understand clonality. We have added mirrored subclonal alleleic imbalance along with findings from previous studies suggesting a complex ITH and subclonal structure.*

*All of these changes are highlighted in the MS (Page 5-6 Line 59-69; Page 9 Line 169-178, Page 19 Line 400-408, 412-424, Page 20-21 Line 430-443, Page 21 Line 447-457, Page 22 Line 465-483).*

"The necessity for an evolutionary model also becomes clear in the authors' discussion about how "independent tumours are quite likely to share CNA at commonly affected loci by chance" (p.7). This does not only affect independent tumours. Parallel and convergent evolution has been demonstrated to be ubiquitous across human cancers and will mislead simple statistical approaches that assume that shared events imply similarity by descent. This issue is even maintained in many evolutionary models which make use of the infinite sites assumption."

*We agree with the comment here, and added few sentences in the earlier paragraphs while describing disadvantages of a simpler approach in Page 5 Line 60-69:*

*"“In many cancer types both parallel and convergent evolution have been detected (Figure 2). In the parallel model polyclonal tumours arise within a patient from the same initiating event but progress along independent but phenotypically similar routes. Breast cancer has been described with a parallel evolutionary model by multiple studies. A convergent model has different initiating cancer cells that acquire the same or highly similar genetic alterations [14, 15]. Germline driven VHL-associated renal cell carcinoma [16] and squamous cell carcinoma [17] were both shown to be convergent, whereby even independent tumours carry similar genetic alterations overall. This can include allele specific chromosomal changes since both tumours evolving in the same host have the same microenvironment and similar selection pressures. Thus, when only lower resolution data is available, any approaches taking into account only overall CN might give a strong clonality signal regardless of the true relationship.""*

"In summary, the review would benefit from restructuring and a clear definition of what its scope is. The authors must reference the evolutionary context more clearly. In addition, some of the "context-specific considerations" are really basic problems of dealing with a certain type of data. For example, point 4) that SCNA events "build on and can obscure initiating breakpoints" (p.20) is solved by event-based evolutionary approaches such as MEDICC, which the authors briefly discuss."

*We have not deleted the context part since the other reviewer found it important, however, while re-structuring the review, we have made it more concise when discussing the tools for lower resolution technologies and the context.*

"As a side-note, with a heavy focus on SCNA analysis, the review does not mention whole-genome doubling events as a key indicator of clonal relatedness, which I consider an oversight and should be addressed. Also, there is no mention of allele-specific vs total copy-number, an important distinction between aCGH data and NGS or SNP array data which enables inclusion of LOH events and parallel evolution and might be useful to mention in the paragraph about LOH events."

*We have now covered the evolutionary models and WGD events together with allele specific events. There are multiple paragraphs added to the latter part of this review and also Figures 2 (models) and 3 (mirrored subclonal AI and parallel evolution).*

"Minor points

The abstract is very short and would benefit from a clearer description of what this review covers"

*We have clarified our scope in the abstract.*

"p.3: the authors state that "Various technologies were employed such as [...] CGH arrays or SNP arrays. In this review we will focus on more advanced technologies [...] (i.e. CGH arrays, SNP arrays)". I assume the first sentence was supposed to name less advanced techniques? Then I would remove the reference to CGH and SNP arrays from the first sentence."

*We have changed and clarified the technologies in the introduction.*

"p.4 - l37: "considered as an event": It is unclear what an event is here, this needs to be defined."

*We have added: considered as a genetic event (gain/loss). Page 4, New Line 36*

"p.5 - l.57: "Thus, clustering together of two tumours from the same patient readily indicates the clonal relatedness": Really the only thing it indicates is that they are similar according to the statistics employed. This similarity might or might not have to do with relatedness (see comment above about evolutionary model, parallel evolution etc.)."

*We have changed this sentence in Page 4 New Line 47: "“Thus, clustering together of two tumours from the same patient indicates the relative similarity of the pairs, but cannot determine the statistical confidence of these relationships and cannot be applied to a single pair alone.”"*

"p.5 - l.61: "(percentage concordance, hierarchical clustering, similarity score)": These are not alternative methods. Percentage and similarity score are both similarity scores, whereas hierarchical clustering is a clustering method which also requires a distance (dissimilarity) measure, typically euclidean (which is also non-evolutionary). Supposedly, concordance and similarity scores were also subjected to some clustering in this study."

*We have now made a concise description of similarity approaches together under one subheading in the MS and left the details of each of these in Table 2, which covers the tools for low depth NGS/array based technologies.*

"p.6 - l.75-80: "Given this high frequency of chromosomal changes, it is possible that many pairs would show clonal relatedness by chance.": they might show similarity by chance which erroneously would be interpreted as clonal relatedness. This is not necessarily a problem of SCNAs as such, but of the method used to infer relatedness (event-based methods are likely to do much better)."

*We agree that it is not an issue of SCNA as such, but more to do with the lack of precision of SCNA detection.*

"p.6 - l.83: Please reference a suitable test dataset if you have one in mind."

*We are not aware of any studies that have undertaken such an analysis (hence why this is considered lacking), but think that it would be a useful control data set to understand the degree to which independent breast tumours may be similar to each other given the germline/environment context. However, in the process of revising the focus of the MS, we have removed this part.*

"p.10 - l173: " (i.e. SNP arrays provide more precise breakpoints than NGS).": That's not true, please remove. Generally NGS data has improved resolution and precision over SNP arrays, and nucleotide-level breakpoints via split-read analysis cannot even be performed with SNP arrays."

*We have removed this part at the request of other reviewers and we agree that high resolution NGS would provide stronger evidence of breakpoints than SNP arrays. Initially this comment was meant for low depth sequencing and hence it created confusion.*

"p.11: The excursion into SCNA segmentation (CBS etc.) seems superfluous."

*We have deleted the segmentation details.*

"p.17 - l.354: Pleas also cite https://pubmed.ncbi.nlm.nih.gov/25293804/"

*This publication is added: Page 19, Line 410*

"p.17 - l.362: PyClone does not only take SCNA input from ABSOLUTE or FACETS. I would remove the specific SCNA callers or alternatively be complete and add at least ASCAT and SEQUENZA and add references for all."

*We have removed the unnecessary details of these tools.*

"p.18 - l.370: Please refer to the latest version and provide the minimum event distance https://www.biorxiv.org/content/10.1101/2021.02.28.433227v1)"

*We have described this tool, MEDICC2, including the MED.*

"p.25 - l.543: I would not agree, in most clinical studies blood is taken as a control tissue, which is fully sufficient for SCNA analysis, does not typically contain many somatic mutations. Further, normal tissues are rarely "enriched with aneuploidies" (depending on the tissue), this is actually a major difference to SNVs (of which they have plenty)."

*While prospective studies do indeed collect blood, this is not the case with retrospective studies that take advantage of archived tissues, which are a valuable source of samples with long clinical follow up. We have added in Page 23 Line 507.*

*"“..yet the feasibility of obtaining germ-line DNA is problematic for retrospective studies using archival tissue samples with long follow up or those collected through routine diagnostic procedures.”"*

*While we agree that somatic SCNA in blood are not a major issue, somatic mutations in blood due to clonal haematopoiesis could be problematic, although unlikely to be shared with a carcinoma. SCNA are rare in normal tissues (although occasionally observed in specific scenarios such as clonal expansion of the ovarian stroma in serous tumours (see Hunter et al, doi: 10.1158/1078-0432.CCR-11-2080), and we have rephrased this sentence to remove the inference that they are “enriched”*

"Figure 1: I don't think I fully understand the Figure, many of the discussed similarity scores are independent of the underlying technology. In particular SNP arrays and WES/WGS are often times exchangeable for SCNA analysis. The figure would also benefit from some visual improvements including a cleaner layout and less text."

*We have replaced Figure 1 with a new figure that better summarises the tools and their application.*

**Second round of review**

**Reviewer 2**

I would like to thank the authors for attempting to address the issues I raised in the first version. Unfortunately, reading the article was still difficult und I still think it needs a substantial amount of work to be useful to a wide audience. The biggest issue is as before with the preciseness of the terminology and the structure. In the introduction it is still unclear what exactly is meant by "clonality". Is the question about a quantitative assessment of how evolutionarily distant two samples from the same tumour are? Or is the question about statistical tests to investigate whether any two samples share a common ancestry, e.g. to distinguish between a recurrence and a secondary primary? Or both? The introduction ll.11ff only talks about "clonal relatedness" and then "clonality". Please let the reader know what you're after. Related, are we talking about similarity between samples? Or clones? This gets mixed up in the review unfortunately and it is a crucial distinction as the "subclonal reconstruction" chapter shows. In the first chapter "Estimating clonality..." the authors introduce SCNAs and in a) describe a method that uses copy-number states on chromosome arms. What really is meant here I believe is "total copy-number" or "total SCNA" in that the total amount of DNA is measured in a non allele-specific way, i.e. without information about e.g. LOH. Total CN is typically what you get from shallow NGS, and array CGH, but not *all microarray* technologies are low resolution. SNP arrays are arguably as high resolution as normal depth NGS. There is also really no question (at least in chromosomally instable tumours) if SCNAs alone are sufficient to estimate clonality. It is really a question about the resolution of the SCNA readout and whether it is allele-specific or not. A case at the end of b) is then made that allele-specificity (it is really haplotype-specificity; phasing is key here, allele-specific SCNAs are not enough) needs to be taken into account to deal with parallel evolution (the cited papers are NGS papers btw). But the article continues with breakpoint-based analysis. In c) LOH is then discussed, and I was expecting this to be a section about allele- and haplotype-specific SCNAs but it wasn't. It talk about the clonality package and the function "LOHclonality()" which comes somewhat out of the blue. I'm sure the reader doesn't know the function and I don't think the function name adds anything. The BAF is an important concept here (see above). I think the first chapter should be restructured and the evolutionary models for SCNA (e.g. MEDICC) could go here instead of at the end of the article. In the next chapter "Statistical approaches..." the authors discuss methods for SNV analysis. The sections about clonal deconvolution at the end of the article would fit in there much better as the current state of the art approaches when dealing with SNV data. Again b) describes a single function from the clonality package. I would suggest talking about the concept, not a specific function. The chapter "Context-specific considerations..." might be interesting but I'm not sure what is discussed is really specific to low-depth single sample NGS or array based technologies (again array-based is not a good term as SNP arrays are really quite high resolution). Altogether maybe it would be better to disentangle methods from sequencing technologies and use a structure like this • Introduction • Estimating similarities from SCNA data ○ from total SCNA ○ from allele- and haplotype-specific SCNA ○ evolutionary methods (MEDICC etc.) • Estimating similarities from SNV data ○ clonality index ○ statistical tests for relatedness ○ subclonal deconvolution (pyclone etc.) ○ evolutionary methods (phlowgs etc.) • Context-specific considerations • Conclusion And then simply discuss in each section for what technologies they might be applicable. Minor points l248: "utlising" => "utilising"

**Authors Response**

**Point-by-point responses to the reviewers’ comments:**

*>>We thank the reviewer for the detailed suggestions for the structure and content of this review. Their time and effort are greatly appreciated and we hope that the current version is acceptable for publication. We have also discussed the manuscript with the Genome Biology editor and incorporated their suggestions. All new headings, subheadings and paragraphs are highlighted in yellow.*

Reviewer #2: I would like to thank the authors for attempting to address the issues I raised in the first version. Unfortunately, reading the article was still difficult und I still think it needs a substantial amount of work to be useful to a wide audience.

The biggest issue is as before with the preciseness of the terminology and the structure.

In the introduction it is still unclear what exactly is meant by "clonality". Is the question about a quantitative assessment of how evolutionarily distant two samples from the same tumour are? Or is the question about statistical tests to investigate whether any two samples share a common ancestry, e.g. to distinguish between a recurrence and a secondary primary? Or both? The introduction ll.11ff only talks about "clonal relatedness" and then "clonality". Please let the reader know what you're after.

*>>We have clarified the content of this review in the abstract and the Introduction in consultation with the editor. We have been specific as to when we are referring to the ancestral relationship between two lesions (clonal relatedness) versus evaluating intra-tumour heterogeneity. All changes are highlighted Line 10-31.*

Related, are we talking about similarity between samples? Or clones? This gets mixed up in the review unfortunately and it is a crucial distinction as the "subclonal reconstruction" chapter shows.

In the first chapter "Estimating clonality..." the authors introduce SCNAs and in a) describe a method that uses copy-number states on chromosome arms. What really is meant here I believe is "total copy-number" or "total SCNA" in that the total amount of DNA is measured in a non allele-specific way, i.e. without information about e.g. LOH.

Total CN is typically what you get from shallow NGS, and array CGH, but not *all microarray* technologies are low resolution. SNP arrays are arguably as high resolution as normal depth NGS.

There is also really no question (at least in chromosomally instable tumours) if SCNAs alone are sufficient to estimate clonality. It is really a question about the resolution of the SCNA readout and whether it is allele-specific or not.

A case at the end of b) is then made that allele-specificity (it is really haplotype-specificity; phasing is key here, allele-specific SCNAs are not enough) needs to be taken into account to deal with parallel evolution (the cited papers are NGS papers btw). But the article continues with breakpoint-based analysis.

In c) LOH is then discussed, and I was expecting this to be a section about allele- and haplotype-specific SCNAs but it wasn't. It talk about the clonality package and the function "LOHclonality()" which comes somewhat out of the blue. I'm sure the reader doesn't know the function and I don't think the function name adds anything. The BAF is an important concept here (see above).

I think the first chapter should be restructured and the evolutionary models for SCNA (e.g. MEDICC) could go here instead of at the end of the article.

*>>We have restructured the review as suggested by the reviewer based on total CN, allele specific CN and evolutionary methods for SCNA instead of focussing on pre/post NGS. We improved the coverage of the underlying concepts rather than specific functions, particularly allele- and haplotype-specific (Lines 160-180, Page 9-10).*

In the next chapter "Statistical approaches..." the authors discuss methods for SNV analysis. The sections about clonal deconvolution at the end of the article would fit in there much better as the current state of the art approaches when dealing with SNV data.

Again b) describes a single function from the clonality package. I would suggest talking about the concept, not a specific function.

*>>We have adjusted the focus of this section to consider the concept, using the function as an example only.*

The chapter "Context-specific considerations..." might be interesting but I'm not sure what is discussed is really specific to low-depth single sample NGS or array based technologies (again array-based is not a good term as SNP arrays are really quite high resolution).

*>>We have moved this part to the end of the MS as suggested by the reviewer and provided additional rationale for including this section (Line 340-345).*

Altogether maybe it would be better to disentangle methods from sequencing technologies and use a structure like this

Introduction

Estimating similarities from SCNA data

○ from total SCNA

○ from allele- and haplotype-specific SCNA

○ evolutionary methods (MEDICC etc.)

Estimating similarities from SNV data

○ clonality index

○ statistical tests for relatedness

○ subclonal deconvolution (pyclone etc.)

○ evolutionary methods (phlowgs etc.)

Context-specific considerations

• Conclusion

And then simply discuss in each section for what technologies they might be applicable.

*>>We have restructured the MS according to the reviewer’s suggestion. We have an extra subheading under Estimating similarities from SCNA data: “Total SCNA approaches incorporating breakpoint locations” according to the editor’s suggestion.*

Minor points

l248: "utlising" => "utilising"

*>>We have changed the spelling.*

**Third round of review**

**Reviewer 2**

I thank the authors for addressing most of my points. I have a few remaining comments:

1. The distinction of parallel vs convergent evolution on page 7 is debatable. I understand it’s taken from Venkatesan et al. 2017 (ref [17]), but from an evolutionary perspective it is inaccurate, and I personally would not advise to propagate this definition further. Also, your Figure 2 is pretty much a copy of Figure 2 in Vekatesan et al., which I would advise against. This is ultimately for the authors to decide of course.

2. Table 1 states that single-cell sequencing does not allow computation of BAF. This implies that single-cell sequencing does not allow for allele-specific copy-number calling. This is not correct, as for example seen in Minussi et al. 2021 (https://www.nature.com/articles/s41586-021-03357-x)

3. I believe all discussed tools and algorithms should be listed in Table 2, not only a subset. For example, MEDICC2 defines a “minimum event distance” between pairs of SCNA profiles and uses Neighbor-Joining to infer relatedness. Also, please cite the current version of the article with a changed list of authors: https://www.biorxiv.org/content/10.1101/2021.02.28.433227v2

Thanks for all your efforts, I hope you found my comments overall useful. Merry Christmas!

**Authors Response**

**Point-by-point responses to the reviewers’ comments:**

Reviewer #2: I thank the authors for addressing most of my points.

I have a few remaining comments:

1. The distinction of parallel vs convergent evolution on page 7 is debatable. I understand it’s taken from Venkatesan et al. 2017 (ref [17]), but from an evolutionary perspective it is inaccurate, and I personally would not advise to propagate this definition further. Also, your Figure 2 is pretty much a copy of Figure 2 in Vekatesan et al., which I would advise against. This is ultimately for the authors to decide of course.

*We think that for the purpose of calling clonality, we find the definitions in Vekatesan et al to be helpful in explaining how independent tumours can appear similar. Therefore, we prefer to retain this as well as the figure. We have acknowledged the inspiration for the lower half of this figure in the legend.*

2. Table 1 states that single-cell sequencing does not allow computation of BAF. This implies that single-cell sequencing does not allow for allele-specific copy-number calling. This is not correct, as for example seen in Minussi et al. 2021 (https://smex-ctp.trendmicro.com:443/wis/clicktime/v1/query?url=https%3a%2f%2fwww.nature.com%2farticles%2fs41586%2d021%2d03357%2dx&umid=763fef3d-660d-4884-8b76-57f3032ac2d4&auth=bb9959a28cb882eca0abc9f5f7e69fc9efeae31d-635a538bd8d4943113c61cbac0fbed5c884d37ae)

*We have amended Table 1, including the reference suggested.*

3. I believe all discussed tools and algorithms should be listed in Table 2, not only a subset. For example, MEDICC2 defines a “minimum event distance” between pairs of SCNA profiles and uses Neighbor-Joining to infer relatedness. Also, please cite the current version of the article with a changed list of authors: https://smex-ctp.trendmicro.com:443/wis/clicktime/v1/query?url=https%3a%2f%2fwww.biorxiv.org%2fcontent%2f10.1101%2f2021.02.28.433227v2&umid=763fef3d-660d-4884-8b76-57f3032ac2d4&auth=bb9959a28cb882eca0abc9f5f7e69fc9efeae31d-279269195754c0eee5fbe219608ea326b56195a3

*We have amended Table 2 as suggested, including the updated reference.*

Thanks for all your efforts, I hope you found my comments overall useful. Merry Christmas!

*Many thanks to the reviewer for their very constructive comments.*
